# Pain Obtunders

**Published:** 1883-04

**Authors:** 


### PAIN OBTUNDERS.

When dentistry shall have discovered and put in use something that shall, without injury to the tooth itself, temporarily suspend sensation in its tissues, the dentist's chair will be robbed of its terrors, and it will be possible to save many teeth that are now allowed to go to destruction. Whether or not such a thing exists, but as yet remains hidden in the womb of time, is a matter for conjecture. Certain it is that no great progress toward a consummation so devoutly to be wished has yet been made. Mysterious preparations, invested according to their sapient discoverers with magical powers, have been loudly advertised, only to disappoint their too credulous purchasers. All kinds of the most dissimilar drugs have been empirically tested with nearly uniform results, and those who have experimented the most persistently have usually returned sooner or later to primal principles, and have repinned their faith to a sharp instrument and a steady hand in the excavation of carious teeth.

Yet there are agents which will at certain times and in some cases materially lessen the amount of pain. If the long list of obtunders were always at hand, it is quite possible that among them might be found something adapted to, and measurably efficient in each case. The worst of the matter would be that, until we know more of the nature and cause of tooth caries, one would be obliged to try each of his preparations in succession, and of course the right one would be the last in the list, while all its predecessors would aggravate the pain instead of decreasing it. Chloroform sometimes quiets and sometimes distracts. Carbolic Acid will at times almost entirely obtund sensitive dentine, and upon the next trial it is found entirely without virtue. Chloride of Zinc has been known to quite subdue an exceedingly tender tooth, while at the next application it raised the patient out of the chair and caused his very toe-nails to quiver in agony.

current of hot air has in one case exercised a very soothing effect, and in the next has resulted in the fracture of nearly every commandment of the decalogue. Just why these varying effects are observed from the same agent no one is able to say, for they follow no definite rule so far as our present knowledge goes. Alkalies are as uncertain as acids, and sedatives are not more reliable than stimulants, so that we long ago ceased our search for the long sought universal obtundant. But during all the heart-breaking vicissitudes of this exasperating experience, one little bottle has retained its position in the operating case, and to it we have returned when human hopes were blasted and the desires of man failed, because in it we have occasionally found relief from the nightmare which pursues us. It contains that local anæsthetic mixture so long known to the medical world, Sulphate of Morphia and Camphor gum in equal proportions. Ground together they mutually deliquesce and form a very convenient preparation, which has the negative virtue of doing no harm, if it accomplishes no good.

